# Plasmon-Enhanced Fluorescence Emission of an Electric Dipole Modulated by a Nanoscale Silver Hemisphere

**DOI:** 10.3390/nano12173070

**Published:** 2022-09-03

**Authors:** Jiangtao Lv, Minghui Chang, Qiongchan Gu, Yu Ying, Guangyuan Si

**Affiliations:** 1 College of Information Science and Engineering, Northeastern University, Shenyang 110004, China; 2 Hebei Key Laboratory of Micro-Nano Precision Optical Sensing and Measurement Technology, Qinhuangdao 066004, China; 3 College of Information & Control Engineering, Shenyang Jianzhu University, Shenyang 110168, China; 4 Melbourne Centre for Nanofabrication, Victorian Node of the Australian National Fabrication Facility, Clayton, VIC 3168, Australia

**Keywords:** fluorescence enhancement, surface plasmon, nanoscale hemisphere

## Abstract

The spontaneous emission of a fluorophore is altered by the surrounding electromagnetic field. Therefore, the radiation of the fluorophore can be engineered by inter-coupling with the nanoscale plasmons. This work proposes a nanoscale hemisphere structure that enhances the electric field and further modulates its effects on fluorophores by adjusting the radius of the hemisphere. A full-wave simulation is carried out using the finite element method, and the radiation characteristics of the nanoscale hemisphere are studied in detail. Compared with free space, the structure has generated significant enhancement exceeding 30. Through curve fitting, the relationship between the enhanced peak wavelength and the radius of the hemisphere is obtained.

## 1. Introduction

Molecular fluorescence is widely used in a variety of applications, such as medical imaging [1], light source manufacturing [2], biological detection [3,4,5], and so on [6]. However, the weak fluorescence signal limits its applications to a certain extent. Spontaneous emission of emitters is considered to be an invariable intrinsic property. Purcell’s precursory research [7], however, led to the realization that the spontaneous emission of a transmitter can be influenced by the state of the electromagnetic field in its environment. The spontaneous emission of the emitter can be engineered by changing its surrounding electromagnetic field [8]. By coupling the fluorophore with plasmonic devices [9,10] and changing the electromagnetic field around the emitter, the spontaneous radiation of the fluorophore can be effectively adjusted to enhance the fluorescence signal intensity and further improve the detection accuracy and sensitivity.

The manipulation of spontaneous emission from emitters has been intensively studied. Because of their unique optical properties, plasmonic devices have become the focus of nanophotonics research community. When the distance between the emitter and the metal structure is close enough, the fluorescence quenching will occur. On the other hand, coupling between the emitter and the metal structure will be weakened when the distance is large [11]. The overlap of plasmon resonance spectra and fluorescence spectra also affects fluorescence enhancement [12]. The structures that have been studied include silver cubes [13,14,15], nanorods [16], and nanospheres [17]. Through theoretical research and experimental analysis, it has been confirmed that these structures can significantly enhance the fluorescence signal of fluorescent molecules. Some researchers have used periodic array structures [18,19] to enhance fluorescence, such as nanohole arrays [20], dual-blade-like shaped arrays [21], and spherical cavity arrays [22], and they all show remarkable fluorescence enhancement effects. A relatively simple chemical method has also been proposed to obtain the self-assembled structures of metal nanoparticles, and experiments have proved that these structures can also improve fluorescence intensity [23,24,25]. In addition to the use of gold, silver, aluminum and other metallic materials [26,27,28], some researchers believe that the absorption loss of metal materials significantly affects the application of surface plasmons, so the use of high refractive index dielectric materials [29,30,31,32] can help achieve notable enhancement effect.

When the light beam shines on the surface of a metal nanostructure, photons interact with the free electrons in the metal, and the free electrons collectively oscillate to produce local plasmons. Surface plasmon resonance enhances the interaction between light field and fluorescent molecules [33], providing a new channel for radiation attenuation. That is to say, the metal nanostructure enhances fluorescence in several ways: (1) the local field is enhanced to increase the excitation rate of fluorescence; (2) one can increase quantum yield to increase the radiation decay rate of fluorescence; (3) one can improve the directionality of fluorescence emission and the collection rate [34,35]. By changing the shape, size, and material of metal nanostructures, their optical properties can be altered to control the effects of metal nanostructures on fluorescent molecules, including enhancement and quenching.

In this work, a metal hemispherical nanostructure is proposed. Monochromatic spot dipole is used instead of a fluorescent molecule and placed near the metal nanostructure to simulate the interaction between a fluorescent molecule and a metal nanostructure, and their fluorescence properties are studied. The finite element method is used to perform numerical analysis on the entire model. The results show that a single nanoscale hemisphere can significantly increase the fluorescence intensity of fluorescent molecules.

## 2. Materials and Methods

**Figure 1:**
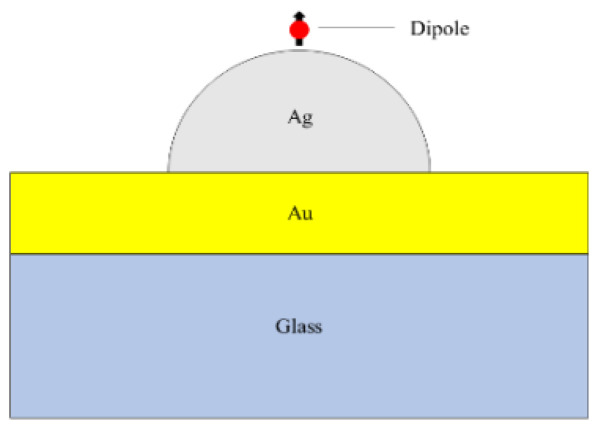
Schematic cross-section of the model under study in this work.

**Figure 2:**
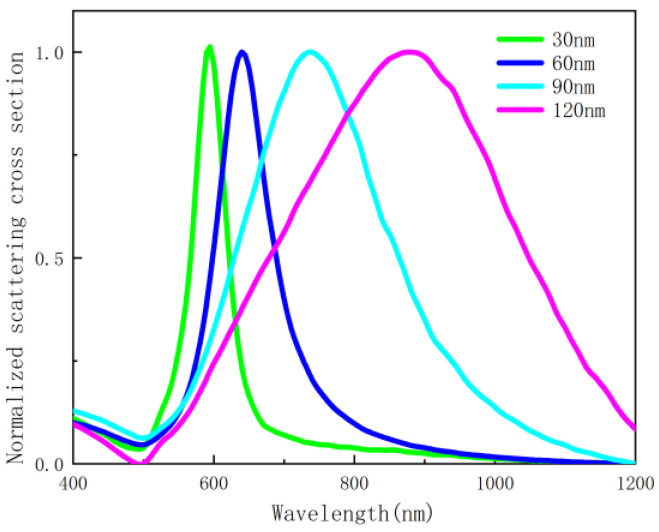
Scattering cross-section spectra of nanoscale hemispheres with different radii.

In this paper, nanostructures composed of silver nanoscale hemispheres and gold films are proposed to be placed on a quartz substrate, as shown in Figure 1. A spherical domain is created around a single silver nanoscale hemisphere with the outermost layer set as a perfectly matched layer to simulate an open boundary. The scattering parameters for gold and silver are derived from the literature [36]. The quartz substrate is assumed to be semi-infinite with a refractive index of *n* = 1.47, which is placed beneath the 50 nm-thick gold film. The emitter is placed directly above the nanosphere. The simulation wavelength range is from 400 nm to 1200 nm. The radius of the hemisphere is set to 30 nm, 60 nm, 90 nm, and 120 nm, respectively. Figure 2 plots the scattering cross-sections of hemispheres with varying radii. The scattered-field formulation is employed, using the analytical solution for an incident plane wave in the absence of the nanoscale hemisphere as the background. Fluorescent molecule is modeled as a monochromatic point-dipole [37].

## 3. Results

In general, the spontaneous decay rate of a dipole is given by

γ = γ_r_ + γ_nr_ (1)

where γ_r_ is the radiative decay rate of the emitter and γ_nr_ is the non-radiative decay. The quantum yield is expressed by the radiative decay rate and the non-radiative decay rate as

Q = γ_r_/γ (2)

The radiation enhancement factor, also known as fluorescence enhancement, is expressed as

F_rad_ = γ_r_/γ_0_ (3)

where γ_0_ is the radiative decay rate of a dipole in free space.

The dipoles in the *x*-axis, *y*-axis, and *z*-axis directions are simulated, respectively. It is found that the dipoles in the *x*-axis and *y*-axis cannot efficiently couple with the plasmon resonance of the metal nanostructure, and their spontaneous emission is weakly affected. The far-field radiation is weak, and the radiation is almost absorbed and dissipated by the metal structure. The dipoles in the *z*-axis direction can be well coupled with the plasmon modes, and the spontaneous emission is greatly affected and enhanced.

**Figure 3:**
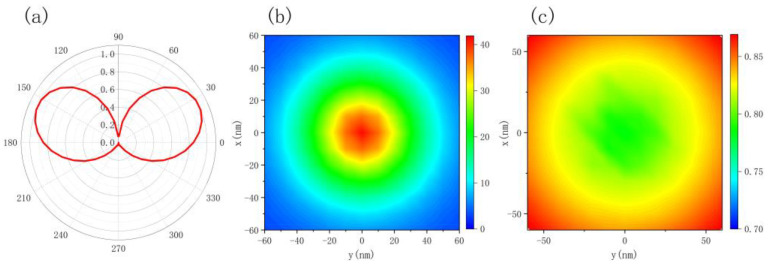
(**a**) Spontaneous emission angular distribution, (**b**) spontaneous emission enhancement distribution diagram, and (**c**) quantum yield distribution diagram when the radius of the hemisphere is 60 nm and the radiation wavelength is 650 nm.

By changing the position of the dipoles placed on a discrete 12 × 12 grid at 10 nm above the nanoscale hemisphere, the spontaneous emission enhancement and quantum yield of dipoles at different positions could be obtained. Figure 3 shows emission characteristic diagrams obtained by full-wave calculations when the emission wavelength is 650 nm and the radius of the hemisphere is 60 nm. Compared to free space, the spontaneous emission rate is significantly enhanced. The calculated spontaneous emissivity enhancement, which depends on the lateral position of the emitter at the surface 10 nm above the nanoscale hemisphere, is more than 40-fold enhanced at 10 nm directly above the nanoscale hemisphere for dipoles in the z direction. At the same time, the quantum yield is kept greater than 0.7. From the simulation of the far-field radiation of the structure, it can be seen from the obtained radiation pattern that the structure has a certain adjustment effect on the radiation direction.

**Figure 4:**
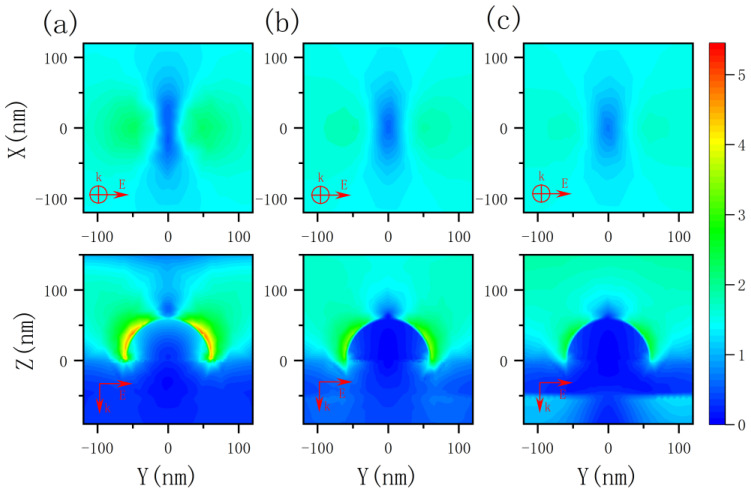
Electric field enhancements ($\frac{\left|E\right|}{\left|E_{0}\right|}$) of the silver nanoscale hemisphere at different wavelengths of (**a**) 400 nm, (**b**) 535 nm, and (**c**) 650 nm. Top row, top view at 10 nm directly above the hemisphere; bottom row, longitudinal section of the structure.

Figure 4 shows the electric field enhancement of the silver nanoscale hemispheric structure at incident wavelengths of 400 nm, 535 nm, and 650 nm, when the radius of the hemisphere is 60 nm. The *k* vector is the incident direction, and the *E* vector is the polarization direction. The results show that the structure can enhance the local electric field at different incident wavelengths to some extent.

**Figure 5:**
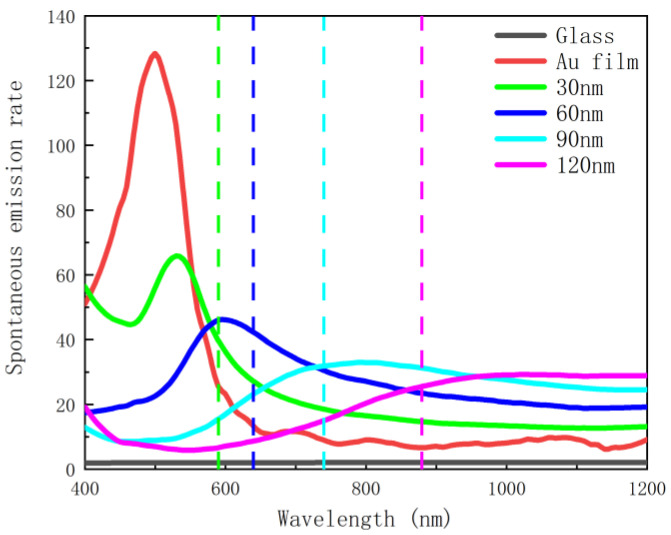
Enhanced spontaneous emission with different hemisphere radii (the dashed lines represent the peak wavelengths of the scattering cross-section).

**Figure 6:**
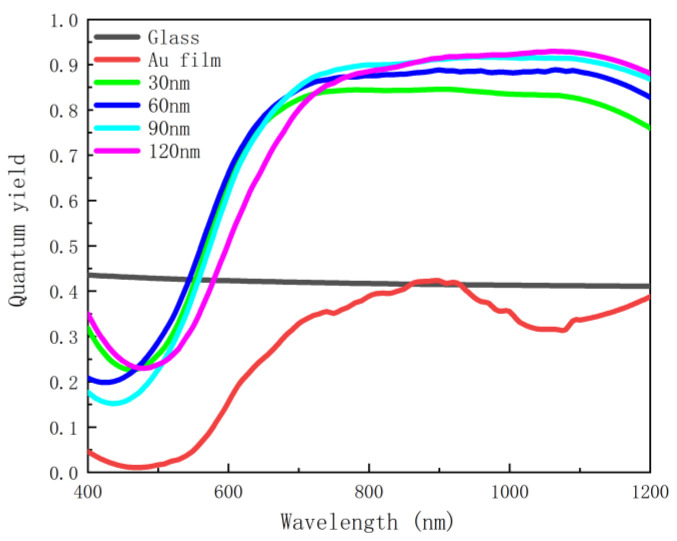
Quantum yield as a function of wavelength with different hemisphere radii.

**Figure 7:**
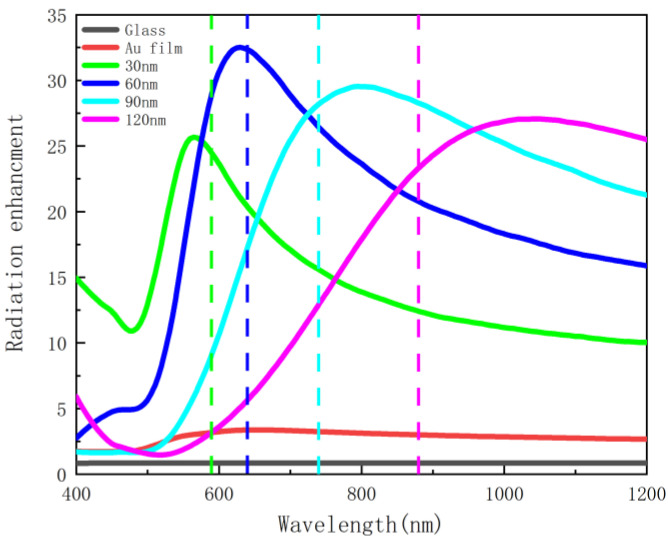
Radiation enhancement as a function of wavelength with different hemisphere radii (the dashed lines represent the peak wavelengths of the scattering cross-section).

It can be seen from both Figure 5 and Figure 6 that, in the absence of the silver nanoscale hemisphere and gold film, the spontaneous decay rate and quantum yield of dipoles are almost unaffected compared to free space, so there is almost no fluorescence enhancement. This is due to the extremely weak coupling between the glass substrate and the emitter, which has limited effect on the emission of the emitter. When the gold film is added, surface plasmon resonance can be generated to couple with the emitter, and the spontaneous emission of the dipole is further affected, and therefore the spontaneous emission is enhanced by about 120 times. However, in terms of quantum yield, almost all the radiation of the dipole is absorbed by the gold film and dissipated as heat, so the gold film has little effect on fluorescence enhancement. After adding silver nanoscale hemisphere, although the enhancement of spontaneous radiation is weaker than that of the gold film, as shown in Figure 7, the absorbed radiation decreases. The results show that fluorescence is significantly enhanced, and an enhanced peak is observed. This is due to the surface plasmon resonance generated by the silver nanoscale hemisphere. Moreover, the local density of states is increased and an efficient coupling with the emitter is generated. The closer the scattering cross-section peak is to the spontaneous emission enhancement peak, the better the radiation enhancement effect. At the same time, with increasing radius of the silver nanoscale hemisphere, the enhancement peak has a red shift, and the enhancement effect changes from weak to strong and then weak, and the enhancement effect is the best in the silver hemisphere with a radius of 60 nm. Moreover, Figure 7 shows that for a hemisphere with a radius of 60 nm; the wavelength corresponding to the maximum enhancement effect almost overlaps with the wavelength of the maximum scattering cross section. Therefore, by changing the radius of the silver nanoscale hemisphere, the wavelength bands of the enhancement peak and the enhancement effects are modulated to enhance the fluorescence in the required wavelength range.

**Figure 8:**
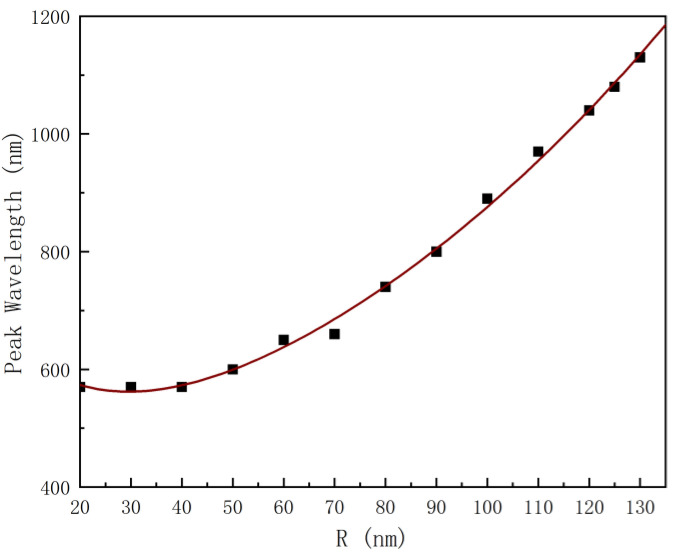
Fitting curve of enhancement peak wavelength and hemisphere radius (R).

**Figure 9:**
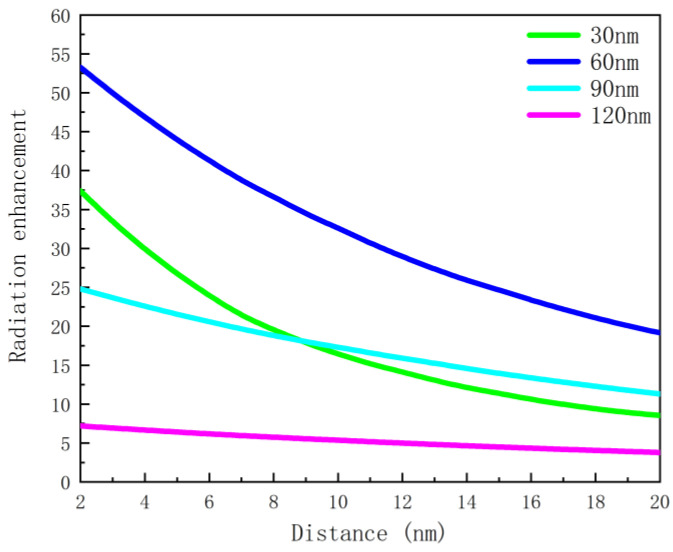
Radiation enhancement as a function of distance at 650 nm emission wavelength.

**Figure 10:**
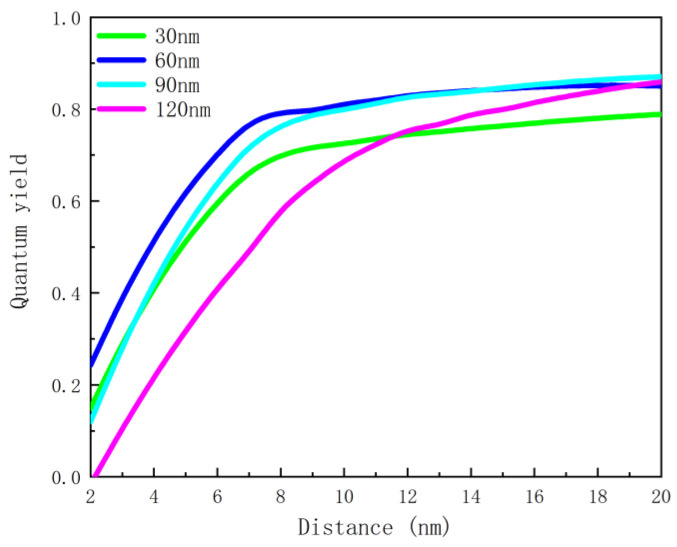
Quantum efficiency as a function of distance at 650 nm emission wavelength.

Figure 8 shows that the wavelength of the enhancement peak is nonlinear and exponential as a function of nanoscale hemisphere radius. The nonlinear equation (y = 345.3 × e^−0.04494x^ + 363.4 × e^0.008754x^, R^2^ = 0.9973) obtained with the MATLAB curve fitting toolbox can be used to determine the radius of the nanoscale hemisphere. The result (RMSE = 12.5808) shows that the method of determining the radius of the nanoscale hemisphere through the required enhancement peak is feasible and has certain application prospects. The effect of the distance between the emitter and the metal nanostructure on the enhancement effect is also investigated. The dipole is placed directly above the metal nanoscale hemisphere, and its fluorescence characteristics are studied by changing the distance between the emitter and the nanoscale hemisphere when the emission wavelength is 650 nm. It can be seen from Figure 9 that the closer the dipole is placed to the metal structure, the more obvious radiation enhancement and the stronger coupling between the dipole and the metal nanostructure can be observed. Meanwhile, Figure 10 shows a lower quantum yield and stronger metal dissipation with closer distances to the metal nanostructure.

## 4. Conclusions

The silver nanoscale hemisphere structure proposed in this work is simulated by the finite element method, and it is confirmed that the structure can effectively enhance the fluorescence. The silver nanoscale hemispheres can boost the radiation by enhancing the electric field, coupling with the dipoles, and increasing the local density of states. With the increase of the radius of the silver nanoscale hemisphere, the radiation enhancement peak shifts to red. In addition, the enhancement effect also generates a peak. When the radius is 60 nm, the enhancement effect is stronger than that of 30 nm, 90 nm, and 120 nm. With different radii, the enhancement effect on different wavelengths appears at peak wavelengths. By fitting the data, it is confirmed that the hemisphere radius has an exponential relationship with the enhancement peak wavelength. Therefore, the desired enhancement peak wavelength can be modulated by changing the radius of the silver nanoscale hemispheres. The closer the dipole is placed to the surface of the silver nanoscale hemisphere (at a certain distance), the more obviously the radiation enhancement effect can be observed. However, from the perspective of quantum yield, the closer the distance is, the greater the metal loss occurs, so radiation is suppressed. The device is expected to be potentially applied in the research fields of biosensing and fluorescence detection.

## Data Availability

Not applicable.
